# Isolation of cells from whole blood using shear-induced diffusion

**DOI:** 10.1038/s41598-018-27779-2

**Published:** 2018-06-20

**Authors:** Jian Zhou, Chunlong Tu, Yitao Liang, Bobo Huang, Yifeng Fang, Xiao Liang, Ian Papautsky, Xuesong Ye

**Affiliations:** 1 Biosensor National Special Laboratory, Key Laboratory of BME of the Ministry of Education, Zhejiang University, Hangzhou, 310027 China; 2 Department of Biomedical Engineering, Zhejiang University, Hangzhou, 310027 China; 3 Department of General Surgery, Sir Run Run Shaw Hospital, College of Medicine, Zhejiang University, Hangzhou, 310016 China; 4 State Key Laboratory of CAD&CG, Zhejiang University, Hangzhou, 310058 China; 5 Department of Bioengineering, University of Illinois at Chicago, Chicago, IL 60607 United States

## Abstract

Extraction of cells of interest directly from whole blood is in high demand, yet extraordinary challenging due to the complex hemodynamics and hemorheology of the sample. Herein, we describe a new microfluidic platform that exploits the intrinsic complex properties of blood for continuous size-selective focusing and separation of cells directly from unprocessed whole blood. The novel system only requires routinely accessible saline solution to form a sandwiched fluid configuration and to initiate a strong effect of shear-induced diffusion of cells, which is coupled with fluid inertia for effective separation. Separations of beads and cells from whole blood have been successfully demonstrated with high efficiency (89.8%) at throughput of 6.75 mL/hr (10^6^–10^7^ cells/s) of whole blood. Rapid isolation of circulating tumor cells (CTCs) from peripheral blood sample of hepatocarcinoma patients is also shown as a proof of principle.

## Introduction

Isolation of cells directly from whole blood with minimal pretreatment is of high demand in liquid biopsy and cytopathology. Minimizing sample preparation not only reduces user intervention and increases reproducibility, but also diminishes labor involved and minimizes process time, as well as lowers testing cost^1–4^. This is especially vital in isolation of rare cells, such as circulating tumor cells (CTCs) from patient peripheral blood^5,6^, where loss of even a single cell can lead to substantial inaccuracies due to rarity of these cells^7,8^. However, direct isolation of target cells from whole blood is prohibitively challenging due to complex hemodynamics and hemorheology.

Many types of microfluidic cell sorting devices have been reported to tackle the challenge of rare cell isolation from blood^9^. External forces, including magnetic^10^, electric^11,12^, acoustic^13^ and optical^14^, have been used in active microfluidic systems for focusing and extraction of target cells from suspensions^15^. Meanwhile, passive systems that rely purely on channel geometry, carrier fluid and cell properties have received attention due to their simplicity and high throughput^15,16^. These include deterministic lateral displacement (DLD)^17,18^, pinched flow fractionation (PFF)^19,20^, hydrodynamic filtration^21,22^, inertial migration^23,24^, viscoelastic focusing^25,26^ and their combinations^27,28^. Additionally, biological affinity has been widely used to target specific cell surface markers and improve selectivity of microfluidic cell sorting^8,29^. While tremendous progress has been achieved, these platforms are not able to work with unprocessed whole blood and generally require a number of sample preparation steps, including lysis of red blood cells (RBCs), immunoselection, or sample dilution. Direct separation of cells from whole blood remains largely unexplored despite of the persistent interest.

The handful of microfluidic devices that can handle whole blood are based on principles of cell margination^30,31^, cross-flow filtration^32,33^, deterministic lateral displacement^34,35^ and immunoselection^8,27^. Additionally, cell deformability coupled with tapered post array^36^ and incorporation of ridges on the top wall of a rectangular channel^37^ have also been exploited to differentiate cell populations passively. However, these approaches suffer from low throughput (0.3–16.7 µL/min) or mediocre separation efficiency (e.g, 27% in continuous^32^ and 72% in discontinuous^33^ cross-flow devices), yet require sophisticated design (e.g., DLD^34,35^ and ridged channel^37^), operational complexity^33,36^, or large device footprint. Hence, these existing approaches are far from practical, and the need for a simple device with high-performance (in terms of efficiency and throughput) still exists.

**Figure 1:**
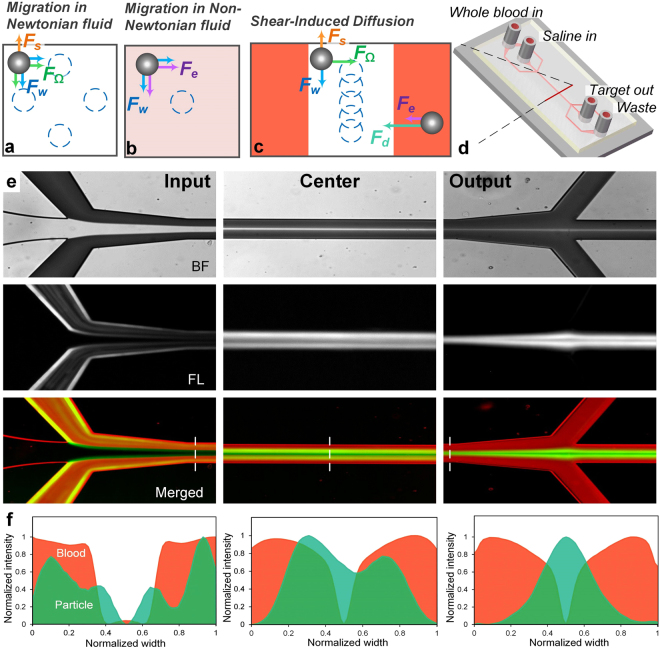
Proposed mechanism and demonstration of bioparticle focusing in whole blood. (**a**) Inertial migration within square microchannel cross-section in Newtonian fluid, with particles migrating toward wall centres under the influence of shear-induced (*F*_*s*_), wall-induced (*F*_*w*_) and rotation-induced (*F*_*Ω*_) forces. (**b**) Migration toward channel center axis dominated by elastic force (*F*_*e*_) in a Non-Newtonian (viscoelastic)fluid. (**c**) Our proposed mechanism of migration in a sandwiched co-flow channel where particles first migrate from blood streams toward the central saline stream under the influence of shear-induced diffusion (*F*_*d*_) and possible elastic force (*F*_*e*_), and subsequently continue to migrate in saline stream toward microchannel center under the influence of inertial forces. (**d**) Layout of our co-flow device, with a focusing length of 10 mm and a cross-section of 100 µm (*w*) × 50 µm (*h*). (**e**) Demonstration of the concept using whole blood sample spiked with fluorescent polystyrene particles (18.7 µm diameter). Bright field (BF) images show flow of whole blood and saline at the channel input, center, and output. Fluorescent (FL) images reveal particle trajectories. Merged images (FL + inverted BF) illustrate preferential focusing of particles (green) from whole blood (red). (**f**) Intensity profiles of blood and particle streams across the dashed lines in (**e**), indicating expanded blood streams and progressively focused particles. The flow rates of whole blood and saline were both 112.5 µL/min.

Herein, we report on a new passive approach for continuous separation from unprocessed whole blood. Our novel separation technique is based on shear-induced diffusion of particles in concentrated suspensions, and is for the first time applied to cell separation from whole blood in a straight, rectangular microfluidic channel (Fig. 1). With a flow of saline solution flanked by sample streams, bioparticles rapidly migrate out of side streams and focus into the cell-free center under the influence of shear-induced diffusion and fluid inertia. Such lateral migration is strongly dependent on cell size. We have successfully demonstrated focusing of polystyrene particles in whole blood within 10 mm downstream length, offering ~90% efficiency. More intriguingly, our throughput remains extremely high (10^6^-10^7^ cells/s or 6.75 mL/h), which surpasses the ultra-fast spiral inertial devices^38,39^. As a proof-of-concept, we successfully separated HepG2 cells spiked in human blood (>89% efficiency) and also isolated CTCs directly from patient blood in our device.

## Results and Discussion

### Human blood and passive focusing

Before we discuss details of our device operation and results, we briefly review blood rheology and its implications on cell focusing. Human blood is a two-phase fluid with various formed elements, exhibiting complex rheological properties. Approximately 45% volume of whole blood is comprised of blood cells, while the rest is plasma which is the aqueous solution with numerous proteins^40^. The majority (~95%) of the suspended blood cells are RBCs. Density of normal whole blood is about 1056 kg/m^3^, which is close to that of water (1000 kg/m^3^) and is primarily determined by plasma and cells^40^. Although plasma shows Newtonian behavior^40,41^, fluid dynamics of whole blood is non-Newtonian, mainly attributed to the dominant population of RBCs (10^9^ cells/mL) and their deformability^40–45^. The mutual interactions of RBCs and interplays with plasma give rise to the viscoelastic dynamics of whole blood. Viscoelasticity can be characterized in terms of Weissenberg number, described as ${\boldsymbol{Wi}}={\boldsymbol{\lambda }}\dot{{\boldsymbol{\gamma }}}$, where *λ* is the characteristic relaxation time and $\dot{{\boldsymbol{\gamma }}}$ is the shear rate^46,47^. In a microchannel with height *h*, $\dot{{\boldsymbol{\gamma }}}=2{{\boldsymbol{U}}}_{{\boldsymbol{f}}}/{\boldsymbol{h}}$, where *U*_*f*_ is the average flow velocity. Both viscosity and elasticity of blood response to fluid shear. At 37 °C, its viscosity is about 4 × 10^−3^ Pa∙s (4 cP) at high shear rate ($\dot{{\boldsymbol{\gamma }}}$> 100 s^−1^) and lowering shear rate increases viscosity significantly which is known as the shear thinning effect^40^.

Viscoelasticity of whole blood suggests a possibility of preferential migration of cells within the complex fluid and subsequent cell separation. Particle (and cell) migration in viscoelastic fluids has been investigated both analytically^48,49^ and experimentally^50–52^. The migration in such flow is primarily subjected to elastic force (*F*_*e*_) and inertial forces in sheared flows (Fig. 1a). The former is described in first and second normal stress differences (*N*_1_ and *N*_2_)^46,50^ and the latter includes mainly shear-induced lift force (*F*_*s*_), wall-induced lift force (*F*_*w*_), and rotation-induced lift force (*F*_*Ω*_)^53^. As illustrated in Fig. 1a, specific focusing positions emerge when inertial forces are dominant at moderate Reynolds number (*Re* = *ρU*_*f*_*D*_*h*_/*µ*, where *ρ*, *D*_*h*_ and *µ* are fluid density, channel hydraulic diameter and dynamic viscosity). On the other hand, particles migrate away from the high to low shear rate region undergoing elastic force (mainly *N*_1_ since *N*_2_ is significantly smaller)^50,54^. Recent works using viscoelastic fluid have shown successful focusing and separation of particulates including polystyrene spheres^46,50^, blood cells^26,55^ and even DNAs^56^ in microchannels.

Despite demonstrations in inertial^16^, elastic^57^, elasto-inertial^25^ or inertio-elastic^55^ systems, whole blood has rarely been directly used in these platforms due to its complex composition and nonlinear rheological properties. Inertial separation is only applicable in Newtonian fluid (*Wi* = 0) and hardly working in whole blood. Focusing of cells using elastic force is ostensibly feasible considering the viscoelasticity of blood. However, the operational condition of negligible inertia (*Re* ≈ 0) imposes minimal flow rate (~µl/hr) and thus reduced shear rate^50–52,56,57^, which could completely ruin device performance. In whole blood, the RBCs aggregate in large numbers and form rouleaux at low shear rate, especially when $\dot{\gamma }$ < 100 s^−1^. The aggregation not only significantly increases the fluid viscosity but also minimizes the intercellular spacings^40,45,58,59^. Escalated viscosity and consequently larger drag force necessitates stronger elastic force for driving cells and without free space cells are difficult to move laterally. Thus, neither of the two approaches alone work for separation of whole blood.

Recent investigations on synergetic interaction of fluid inertia and viscoelasticity (*Wi* > 0, *Re* > 0.1) suggest potential focusing of bioparticles within blood flow, considering the distinctly intriguing properties of RBCs and blood. Mildly increased inertial force (typically 0.1 < *Re* < 10) could effectively eliminate the focusing positions near four corners in a square channel^46,50^. As shown in Fig. 1b, wall induced lift force (*F*_*w*_) becomes sufficiently strong to repel particles inward and they subsequently focus in the channel axis under influence of the elastic force (*F*_*e*_)^46^. The elevated shear rate (>10^3^) as indicated by *Re* helps disaggregate RBC rouleaux and thus reduce blood viscosity (complete dispersion of RBC aggregate occurs when $\dot{\gamma }$ > 200 s^−1^)^58,59^. Furthermore, deformation of RBCs and formation of RBC layers at high shear rate diminish effective volume fraction. This creates additional free space among cells^40,45,58,60^, reduces fluid viscosity and results in a Newtonian-like behavior, which leads to a possibility of lateral migration of cells subjected to both inertial and elastic forces.

### Particle migration in blood flow

**Figure 2:**
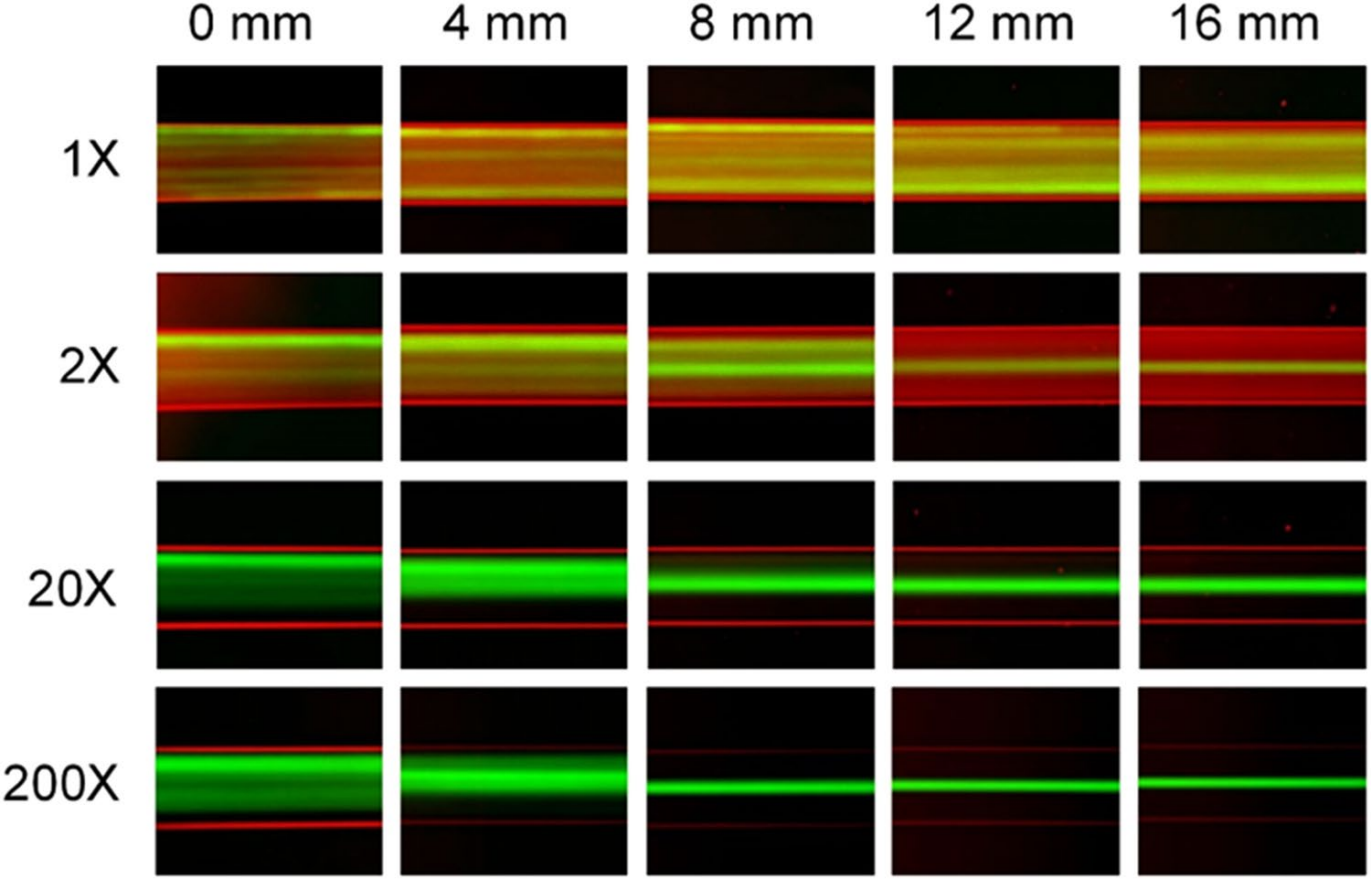
Focusing dynamics of beads spiked into a whole blood at various dilution factors. Images illustrate particle trajectories (green) at successive downstream positions vs. various blood (red) dilution factors in a low AR single flow channel.

When particles are spiked into whole blood, no discernable migration takes place, despite the lateral migration expected due to the synergetic interaction of the inertial and elastic forces. Figure 2 illustrates a straight rectangular microchannel with 100 µm (*w*) × 50 µm (*h*) cross-section. Whole blood spiked with 18.7 µm diameter fluorescent particles was pumped at 225 µL/min (corresponding to *Re* = 50 in terms of Newtonian water flow^53,61^). Considering the Fåhræus -Lindqvist effect, the apparent viscosity of the whole blood was estimated as *µ* = 3.3 cP^40,59,62^ and hence the *Re* was estimated as *Re* = 15. While this is well within the optimal range for inertial migration (10 < *Re* < 100)^53,61^, no migration of fluorescent particles was observed throughout the 24-mm long channel. The most likely explanation for this is the insufficient interstitial space for particle migration due to the high concentration of RBCs.

Results from dilute blood revealed the possibility of more complex force fields that could contribute to particle migration (Fig. 2). Particles in blood diluted even only 2× were successfully focused into a single stream at 12 mm downstream. Analysis of the intensity profiles reveals that tight focusing was reached at 20 mm downstream (Figure S1c). We note that 2× dilution halves the hematocrit (Hct = 22.5%) and viscosity (*µ* = 1.7 cP^40,59,62^) and also modifies the flow to *Re* = 30. While higher *Re* indicates larger inertial force, the focusing pattern differs from that in a Newtonian fluid (Figure S1). Particles achieve complete focusing in Newtonian fluid at 8 mm downstream length (focusing length, *L*_*f*_), which is much shorter than *L*_*f*_ in the 2× diluted blood. Increasing the dilution factor decreases the focusing length, with minimal *L*_*f*_ approaching 8 mm for 200× dilution (Fig. 2)^63^. These results suggested that particle migration in 2× diluted blood is subject to a more complicated force fields, such as inertial, elastic, drag forces and resistance due to interaction between particles and blood cells.

Inspired by the particle migration in blood diluted 2×, we designed a co-flow system to achieve focusing of larger particles from whole blood (Fig. 1c,d). Untreated whole blood was introduced to form two side streams in the main channel, separated by a stream of saline solution. While densities of the adjacent fluids were matched, such configuration created gradients of both cell concentration and viscosity across the two interfaces, higher in the blood streams and lower in the saline stream. These sharp gradients coupled with shear rate in the channel flow can lead to a strong shear-induced diffusion of particles and cells^64^, which may result in an effective and fast mixing of blood and saline. We first hypothesized that, if the flow rates of whole blood and saline buffer equals each other, such mixing may help to establish a similar rheological and flow conditions to those in the 2× diluted blood, permitting lateral migration and focusing of larger cells and particles. Indeed, our preliminary results in Fig. 1b show both rapid mixing of blood cells and successful focusing of 18.7 µm particles from whole blood (Hct = 45%) within 10 mm downstream length, which is even shorter than the *L*_*f*_ in 2× diluted blood.

### Migration due to shear-induced diffusion

The observed fast and size-dependent focusing of particles can primarily be attributed to the collective effect of shear-induced diffusion and fluid inertia. Since blood cells were not fully mixed with saline solution while larger particles were focusing, simple dilution mixing (aforementioned hypothesis) cannot explain the observed phenomenon. In fact, particles in concentrated suspensions are known to undergo complex interactions that give rise to net lateral migration in shear flows^64,65^. This is termed shear-induced diffusion. In our device, the migration observed in the side streams of whole blood could be dominated by the shear-induced diffusion due to the unmatched concentrations and viscosities between adjacent fluid layers in the sheared flow. Leighton and Acrivos^64^ have shown that lateral drift of diffusion is mainly as a result of irreversible interaction among cells in a concentrated suspension. In the direction normal to the plane of shear, the effective diffusivity (*D*) of particles relates to shear rate, particle diameter, concentration (*ϕ*) and viscosity and can be expressed as^64^,

1 $$D=K\frac{{\varnothing }^{2}}{\mu }\frac{d\mu }{d\varnothing }\dot{\gamma }{a}^{2}$$

where *K* is a dimensionless coefficient showing weak dependence on particle concentration (*ϕ*). This is equivalent to volume fraction for particle suspension or hematocrit (Hct) for blood sample. The diffusion directs from blood layer (high concentration, viscosity and shear rate) to saline buffer (low concentration, viscosity and shear rate). As indicated by equation (1), the diffusivity (and thus lateral diffusion velocity) is strongly dependent on concentration and particle size as well as shear rate. Essentially, this size-selective shear-induced diffusion promotes particle lateral migration in our co-flow device, leading to a faster focusing as compared to single flow of two-fold diluted blood (Fig. 2).

We observed that the shear-induced diffusion did not effectively promote particle focusing in whole-blood only flow (Fig. 2). This could be mainly attributed to two factors that suppress the hydrodynamic diffusion. First, the velocity profile of whole blood flow in a microchannel is blunter than normal Poiseuille flow due to the shear thinning effect^66^. Such blunt profile produces relatively flat shear rate across the most of channel width. Secondly, no established viscosity and concentration gradients exist to amplify the effect of shear-induced diffusion in single whole-blood flow.

While concentration gradient enhances shear-induced diffusion, it also facilitates the general diffusion of cells into saline layer, which is known as Brownian motion. However, Brownian motion cannot be the driving factor in our device. On one hand, migration dynamics observed in our device differs from Brownian motion. According to Stokes-Einstein Equation (*D*_*0*_ = *k*_*B*_*T*/(*6πµa)*, where *k*_*B*_ is Boltzmann’s constant and T is absolute temperature), diffusivity *D*_*0*_ of spherical particles due to Brownian motion is inversely proportional to particle diameter. Hence larger particles shall migrate slower than smaller ones, which contradicts our observation where 18.7 µm particles reached the centerline much faster than RBCs (6–8 µm). Further, particle motion in our flow configuration is in fact dominated hydrodynamically. Brownian motion is negligible as compared to shear-induced drift, as indicated by the dimensionless Péclet number ($Pe=\,\dot{\gamma }{a}^{2}/{D}_{0}$)^67^. Brownian motion predominates particle behavior when Pe < 1. Péclet number within our device is on the order of 10^5^–10^8^ depending on particle/cell size, which is excessively larger. As a result, the effect of the general diffusion exerts minimal effect on particle lateral migration in our case.

The other main driving force stems from fluid inertia. Particles flowing in Newtonian dilute suspensions are subjected to inertial forces when Reynolds number is not vanishing^68^. These forces are strongly size-dependent^53^: *F*_*w*_ ~ *a*^3^, *F*_*s*_ ~ *a*^2^, and *F*_*Ω*_ ~ *a*^3^. Wall-induced lift force (*F*_*w*_) always deflects particles toward channel center and shear-induced lift force (*F*_*s*_) drives particles in the opposite direction. Rotational force (*F*_*Ω*_) drives particles to their equilibrium positions when they are in proximity of walls^53,69^. Inertial forces are the dominant driving factors that entrain particles and cells once they enter the Newtonian saline stream in our sandwiched flow. Although inertial forces also exist in the whole blood streams (Re ≈ 15), they contribute little on particle lateral migration. While the high concentration of RBCs is favored for shear-induced diffusion, the strong cell-cell interaction hampers the effect of inertial forces, which is evidenced by the observation of no discernable migration in whole blood (Fig. 2). In fact, cell-cell interaction is generally avoided in inertial focusing devices by limiting cell/particle volume fraction (*ϕ*) to be less than 2%^63,70,71^. Inertial effect diminishes sharply at higher *ϕ*^63,72^. Little effect of inertia in concentrated suspension was also pointed out by Madanshetty and Nadim^73^ in their experimental investigation of shear-induced diffusion (*ϕ = *25%). Nevertheless rotational force might have some effect on migration due to the strong shear thinning effect^74,75^ displacing particles to sidewalls and to the formation of RBC layers^45,59^ acting as transient walls in whole blood. This is partly evidenced by the particle migration within 2×-diluted blood streams (Fig. 2).

Since whole blood is essentially non-Newtonian, it is possible that elastic force may affect the migration. Although blood plasma is believed to be Newtonian^40,44^, the addition of deformable RBCs gives rise to non-linear behavior of whole blood. The elastic component of blood is too small to be measured when Hct < 20%. At higher hematocrit the elasticity of blood increases sharply scaling with the third power of Hct^76,77^. However, this viscoelasticity could still be very weak for whole blood considering typical value of Hct = 45%. Should the elastic effect influence particle migration, it could only contribute positively. On one hand, the elastic force is also strongly size-dependent (*F*_*e*_ ~ *a*^3^). On the other hand, while shear-induced lift force counteracts both elastic and wall forces vertically in our low AR channel, forces in horizontal direction are essentially in harmony, directing from sidewalls to channel center (Fig. 1c).

In summary, particle migration within side streams of whole blood is dominated by shear-induced diffusion and inertial force is the primarily driving force in saline stream for particle focusing from whole blood. Owing to the strong size dependence of both shear-induced diffusion and inertial effect, larger particles in whole blood migrate much faster than smaller RBCs and achieve tight focusing in the middle of channel width before being significantly contaminated by the RBCs. These two effects work in tandem to transport larger particles out of the blood stream.

### Device optimization

**Figure 3:**
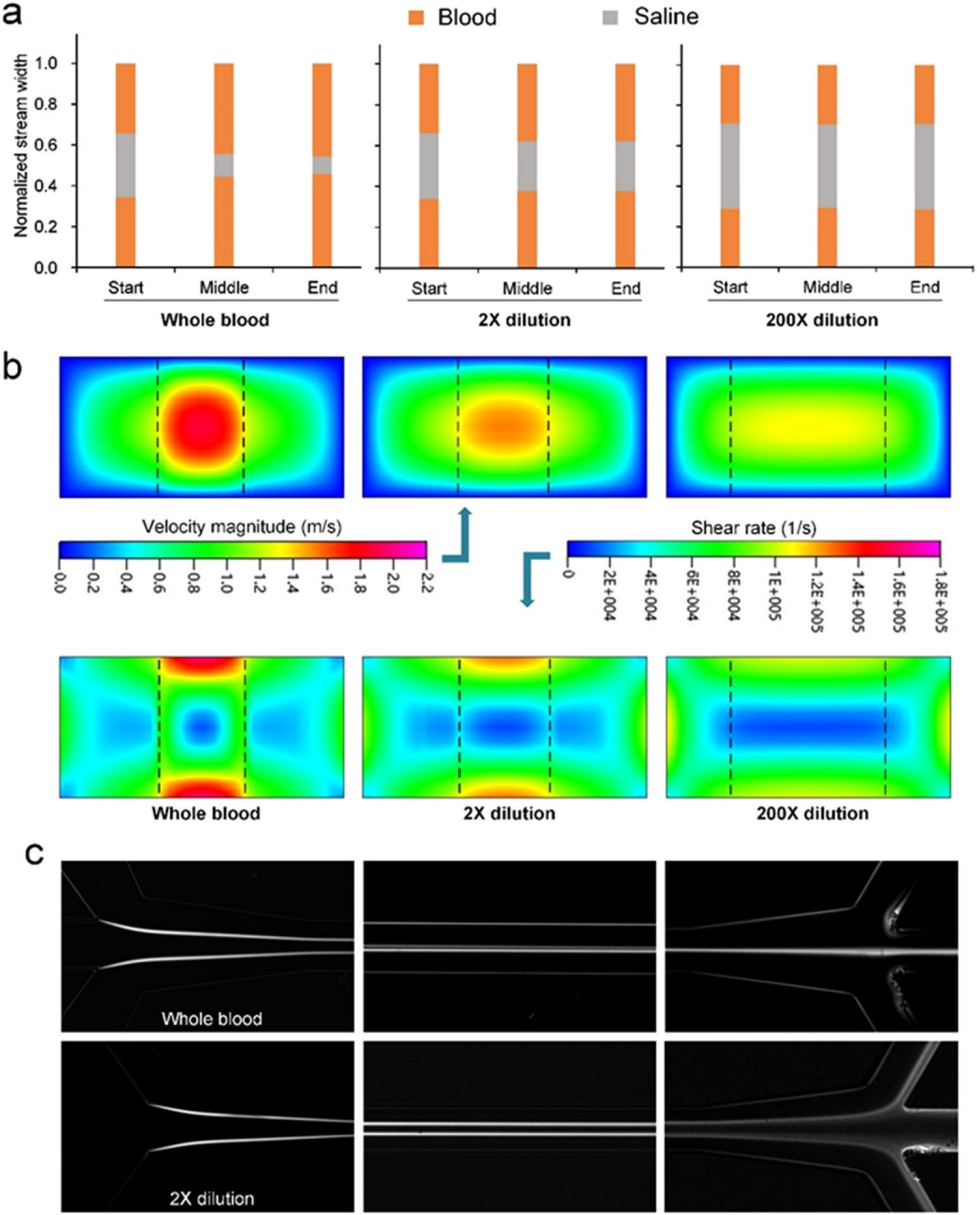
Dynamics of whole blood flow in the co-flow microfluidic systems. (**a**) Shear induced diffusion of blood cells leads to expansion of blood streams. (**b**) Modified velocity and shear rate profiles within channel cross-section at start position obtained from numerical simulation (ESI Group, ACE+). Dashed lines represent the interfaces of the two adjacent fluids. (**c**) Interface between blood and saline streams. Channel length was 10 mm. Each micrographs was generated from the standard deviation of 500 bright field images.

Successful demonstration of particle focusing and separation from whole blood led us to explore the dynamics of bioparticle motion within our co-flow system to improve focusing quality and isolation performance (Fig. 3). According to the merged images and the intensity profiles in Fig. 1b, particles collected from the inner outlet (target outlet) accompanied considerable number of RBCs, downgrading the separation purity. Since RBC contamination primarily arises from the strong shear-induced diffusion, careful tuning of RBC cross-stream diffusion can be an effective way to improve the separation quality. According to equation (1), we could reduce the RBC diffusion by decreasing either cell volume fraction (Hct) or shear rate as the diffusion coefficient is strongly dependent on cell concentration (*D* ~ *ϕ*^2^) and scales with shear rate ($D\, \sim \,\dot{\gamma }$). While reduction of shear rate helps inhibit cell diffusion, it can significantly suppress inertial forces (${F}_{i}\, \sim \,{\dot{\gamma }}^{2}$)^53^ which is one of the main driving force. In addition, both approaches could adversely affect the throughput. Thus, the former is preferred since it is more effective.

We first examined the lateral diffusion of RBCs downstream the channel at three cell volume fractions (Fig. 3). At Hct = 45%, the two blood streams expanded into the central saline layer drastically, leaving only ~8 µm spacing between them at channel end (10 mm downstream) when the total flow rate was 225 µL/min and flow rates were equal for blood (*Q*_*b*_) and saline solution (*Q*_*s*_). Two-fold dilution of blood in saline exhibited remarkable improvement in terms of confinement of RBCs as the spacing tripled (~25 µm). In both cases, blood streams expanded nonlinearly with respect to downstream length, fast in the first half and slowly in the other half as shown in Fig. 3a–c. The observed reduction of lateral diffusion is indeed implied in equation (1) since cell concentration continuously decreases as diffusion progressing downstream the channel and cells experience larger shear rate when they are closer to side walls at the first half channel. Excessive dilution of blood (200×) led to the minimized lateral diffusion and utmost confinement of RBCs within side streams indicated by the unchanged stream widths throughout the channel. The variations of initial stream fractions for the three cases were mainly resulting from different fluid viscosities of blood streams.

In addition to the evolution of stream fractions, blood dilution also modified the patterns of velocity and shear rate profiles at identical flow rates (Fig. 3b). For excessive dilution (e.g., 200×), the viscosity of side streams closely matched with saline solution and both the velocity and shear rate profiles was continuously and smoothly distributed within channel cross-section, forming a large rectangular region (approximately 65 µm × 15 µm) of low shear rate. While such shear rate pattern is preferred in inertial focusing where particles equilibrate in the two positions near walls^53^, it is not desired for migration due to shear-induced diffusion, as particles would focus into a broad band instead of a tight streak. For less diluted blood, the increased viscosity difference between adjacent fluids rendered to the distorted velocity profile with elevated velocity in saline stream and broke the previous low shear rate region into three sections (Figs 3 and S2). For undiluted whole blood, the two side regions tended to vanish, with the lowest shear rate region restricted to a small blue spot (~13 µm in diameter) in the center. Such a small region is ideally preferred for tight focusing of bioparticles dominated by shear-induced diffusion which drives particles to region of low shear rate. Nevertheless, this focusing spot was bounded by a high shear rate blockade (the green annulus in Fig. 3b), which could otherwise prevent migration of bioparticles from the side streams theoretically. Considering the remarked concentration gradient across the blockade and the development of flow downstream, cells could still migrate into the saline stream. Thereafter, blood cells along with plasma rapidly diffuse into the saline layer as evidenced by the evolution of intensity profile of blood streams in Fig. 1b(v–vii). The fast-diffusive migration increases the viscosity of center layer, lowers the viscosity gradient across the fluid layers and thus reshapes the velocity and shear rate profiles downstream. Subsequently, inertial force, potentially also with elastic force, drives the bioparticles toward the channel center.

**Figure 4:**
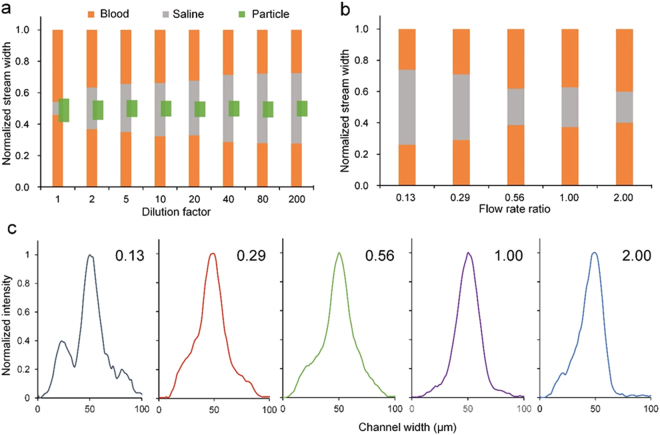
(**a**) Measured fractions of stream widths (blood, saline and particles) as a function of blood dilution factor at the channel output where channel width expanded to 192 µm. (**b**) Fluid stream widths as a function of flow rate ratio (*α* = Q_b_/Q_s_) in the co-flow channel and the corresponding intensity profile of spiked particles at channel end (10 mm). 2× diluted sample was used here. Note that the total flow rates were 225 µl/min for all flow rate ratios other than 0.56 in part (**e**,**f**), where total follow rate was 312.5 µl/min. All channel cross-sections were 100 µm (*w*) × 50 µm (*h*).

We aimed to achieve both tight focusing stream and broad central buffer region for high quality separation. The buffer region is indicated by the spacing between the two sharp boundary lines in Fig. 3c. While particles could achieve single-file focusing in whole blood, the separation spacing was minimal. The 2× dilution serves to significantly enlarge the spacing for easy separation. As dilution factor increased, the separation spacing expanded and meanwhile the focusing quality improved, as evidenced in the decrease of particle stream width (Fig. 4a). The measurements were taken right at the output trifurcation (end of expansion with width $\approx$ 192 µm). Particle stream achieved tight focusing at 5× dilution (Hct = 9%) but the fractions of blood streams remained unstabilized until dilution factor reached 40× (Hct = 1.1%, Figure S3), which is consistent with our previous measurements^63^. Despite better focusing quality and larger separation spacing, the throughput markedly decreased at larger dilution factor. With the decrease of blood concentration, the dynamics of particle migration also alters. For whole blood (Hct = 45%), lateral migration of particles is mainly under the control of shear induced diffusion and inertial forces working in tandem. When cell concentration significantly reduces at hundred-fold dilution, inertial forces increase considerably (reduced viscosity augments Reynolds number) and become dominant in particle transversal motion and the effect of other factors minimizes.

The other approach to increase the separation spacing is the flow rate ratio (*α* = *Q*_*b*_*/Q*_*c*_) of blood and saline solution. We used blood sample diluted twice to investigate the effect of flow ratio *α* (Fig. 4b) at total flow rate (*Q*) of 225 µl/min. We could not plot the particle stream width due to the existence of multiple peaks (Fig. 4c). Although the separation spacing expanded at smaller flow ratio (*α* = 0.13 and *α* = 0.29), particles were not fully focused and multiple streams were observed. While longer channel may help to achieve full focus in this case, the throughput was reduced considerably. In accordance with the intensity profile, balanced flow rates (*α* = 1) were beneficial as tight focusing is achieved in the channel center. Further increase of flow ratio only showed slight decrease in the spacing between the two blood streams, and the focusing quality slightly downgraded as well. We also tried to enhance the input flow rate to ~312 µL/min and set *α* = 0.56. We found the separation spacing was comparable to that when Q = 225 µL/min and *α* = 1, since higher shear rate at larger flow rate strengthened cell diffusion according to equation (1), and the particles were focused but not completely as shown in Fig. 4c. As a result, equal flow rates shall be the optimal in our short channel.

### Separation of cells from whole blood

**Figure 5:**
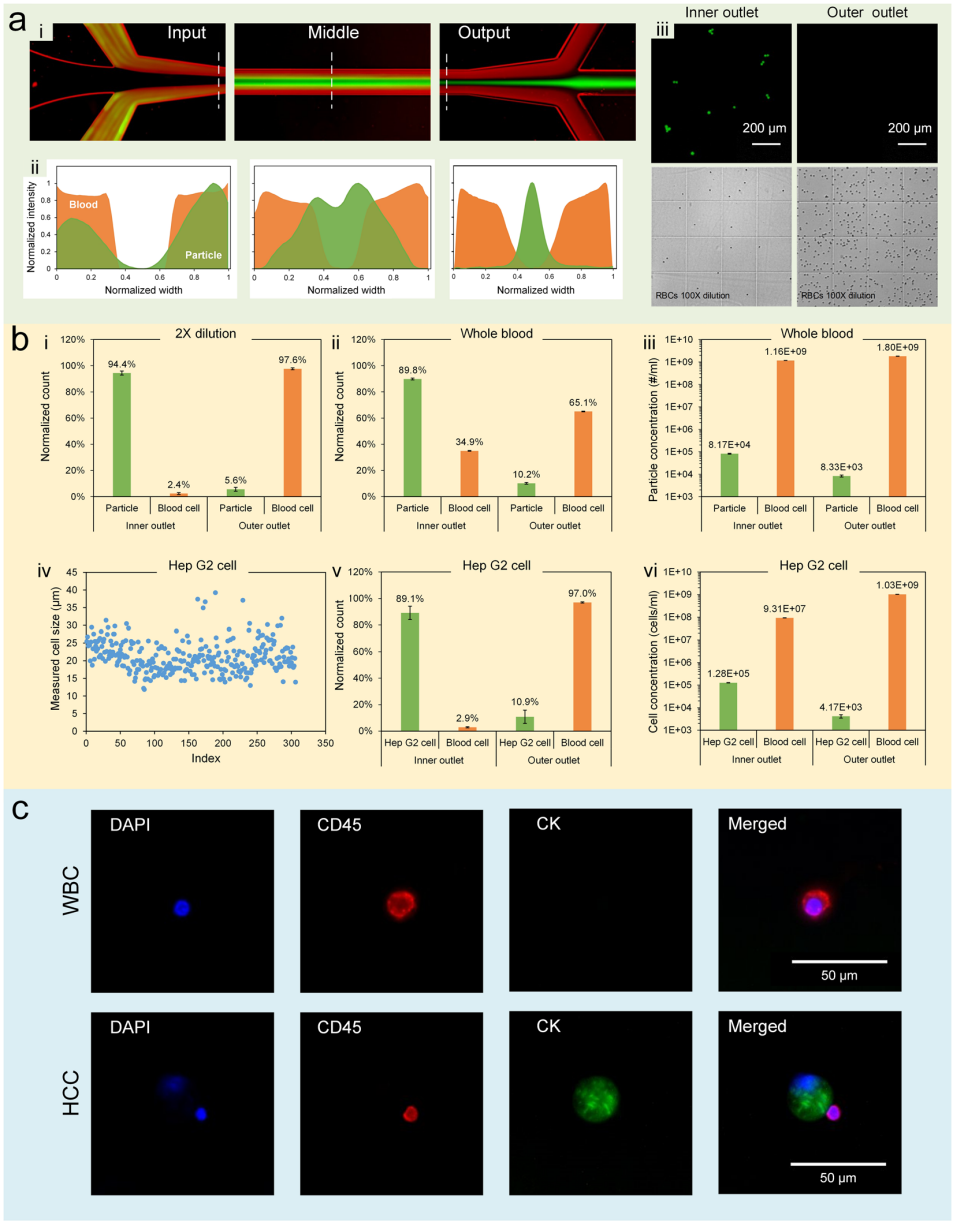
Separation performance of our co-flow system. (**a**) Focusing and separation of fluorescent particles (green) using a 2× dilution blood (red): (i) overlapped pseudocolored images indicating a high-efficiency separation; (ii) the corresponding intensity profiles across the dashed lines in part (i); (iii) micrographs showing the separation outcomes. (**b**) Quantitative results of separation performance using whole blood and 2× dilution blood spiked with particles and Hep G2 cells. Blood cells instead of RBCs were used here since WBCs were also present in the sample. However, the percentage of WBCs was less than 1%. Error bars represent standard deviation of three tests. (**c**) WBC and hepatocarcinoma cell (HCC) extracted and identified from clinical sample (2× dilution) in our system. Error bars are standard deviations of three tests. All flow rates were 225 µL/min with *α* = 1.

As a model system, we evaluated the performance of our co-flow system using polystyrene particles as surrogates for cells. We firstly spiked fluorescent particles into blood sample and measured the outcomes (Fig. 5). The optimal flow condition was chosen (*Q* = 225 µl/min and *α* = 1). First, 18.7 µm diameter particles were spiked into a 2× diluted blood and injected into our channel. The central sharp stream (green) at output shows well focused particles (stream width = 24 µm) and the blood cells (RBCs) were confined within the side streams, giving 13 µm gap at each side of the particle stream and permitting an easy separation of larger targets. The corresponding intensity profiles also reveal a considerable improvement in both focusing quality and separation spacing for 2× dilution as compared to undiluted whole blood (Fig. 1e). We noticed in both cases particles were rapidly entrained into two confluent streams at half channel length, and then merged into one stream centered channel width. The adverse effect of shear-induced diffusion on RBC contamination was well remedied, as uncovered by the intensity profiles of blood streaks (Fig. 5a). The micrographs of the collected samples from inner and side outlets imply high separation efficiency and effective removal of RBCs in the target outlet.

Quantitative results of collected samples confirmed the high-profile performance (Fig. 5b). 94.4% of fluorescent particles were collected from the inner inlet and 97.6% of RBCs were removed. Blood cells in Fig. 5b refer to RBCs as the quantity of WBCs was thousand times lower than RBCs. Our system was highly reliable as indicated by the small error bars. Since separation efficiency is defined as the ratio of target count in central outlet over the total count of target in both outlets, it equals to the normalized count in Fig. 5b. Hence, the separation efficiency was 94.4% for 18.7 µm particles in a blood diluted two times. To compare the separation efficiency of whole blood processing, we also introduced the undiluted whole blood spiked with the same number of particles into the device. It turned out that the separation efficiency remained highly promising (~90%). But the rejection rate of RBCs markedly decreased (64.8%) for inner outlet (target outlet). This was expected according to the strong influence of shear-induced diffusion in whole blood. While these separation efficiencies are comparable to recently work by Geislinger *et al*.^78^ using pinched flow fractionation in viscoelastic fluid, our throughput overpowers their system (20 µl/hr) by a factor of 337. Our separation scheme also outperforms existing systems based on cell margination^30,31^ and cross-flow filtration^32,33^ and is simpler than DLD^34,35^ and other^36,37^ devices. While DLD device from Austin’ group^79,80^ showed comparable efficiency (~86%) with very high flow rate, their system requires 5–20 times dilution and multiple additives to prevent clogging. Similar dilution factor is also necessary for inertial microfluidic devices regardless of various geometries^39,63,81^. Recently, Lee *et al*.^82^ reported separation of MCF-7 cells from whole blood in their contraction-expansion device, showing a slightly better efficiency. Nevertheless, their sample throughput (5 μL/min) was much lower than ours (112.5 μL/min).

### Isolation of cells from clinical sample

To validate the fidelity of our system for cell separation from blood, we utilized human blood spiked with Hep G2 cells to demonstrate the extraction of circulating tumor cells which are typically larger than blood cells. Hep G2 cell line is a perpetual cell line which was derived from the liver tissue of a male with hepatocarcinoma. The size distribution was measured and plotted in Fig. 5b(iv). ~ 90% of Hep G2 cells were found larger than 15 µm. We spiked 1 ml Hep G2 cell suspension (initial concentration 1.7 × 10^5^ cells/ml in PBS solution) into 1 ml human whole blood from venipuncture and carefully stirred to allow adequate mixing. The mixture was processed through our device at room temperature for ~14 min. The Hep G2 cells were pre-stained before mixing for easy counting. Our results confirmed the high separation efficiency for cells as well (89.1%), which is well-matched with size distribution of cells. The slightly decreased rejection rate of RBCs was primarily due to the variation of fluid resistances of outlet branch among different fabrication batches. Based on the concentrations measured in Fig. 5b(vi) and the collected volumes, we estimated the recovery rate of 82% (the ratio of target cells collected in inner outlet over total cells injected). Considering the broad spectrum of Hep G2 cell size (10~45 µm) and the cutoff size (~15 µm) of our channel (in the case of inertial focusing)^63^, this recovery rate could still be very high and was comparable to that (85%) in the spiral devices^38,39^, where blood lysis was required and the separation efficiency was lower.

As a proof of concept, we also demonstrated isolation and identification of circulating tumor cells (CTCs) from peripheral blood donated by patients with hepatocarcinoma (TNM staging: T3N0M0). 1 ml blood was obtained through venipuncture and collected in Vacutainer tube coated with EDTA. Hepatocarcinoma cells (HCC) are typically larger than most of blood cells and the CTCs in these patient bloodstreams can be even fewer compared to other cancer types (e.g., breast cancer)^83^. Hence it is more challenging to separate CTCs for these patients. We utilized a post array right after the target outlet to immobilize suspected CTCs (Figure S3) and to minimize the chance of cell loss due to otherwise off-chip cell collection. After a single run, we successfully extracted and identified one CTC from 1 ml blood samples by onchip immunostaining. While white blood cells were CD45^+^ (Red), CTC was identified by pan-cytokine (CK, green). The identified cell was 23 µm in diameter (Fig. 5c). Two additional tests using the same protocol were carried out in two days and each detected one CK^+^CD45^−^ cell, with diameters of 18 and 26 µm, respectively. Some leukocytes were also observed in the target outlet (Fig. 5c) due to their broad size variation. The cutoff size of our chip was about 15 µm and thus WBCs sized larger than that also entered the immobilization chamber. Observing these results, we believe our approach could be a powerful alternative in isolation or depletion of larger targets from highly concentrated sample.

## Conclusions

In conclusion, we have successfully demonstrated a new scheme for continuous focusing and separation of bioparticles directly from human whole blood. Our system takes advantages of shear-induced diffusion that utilizes the intrinsic complex nature of blood, and couples it with migration due to inertial force for size-based separation. A separation efficiency of ~90% was achieved in whole blood spiked with microparticles at blood flow rate of 6.75 ml/hr, with a throughput up to 10^7^ cells per second which is even higher than the ultra-fast spiral systems. In a 2× diluted sample, higher separation efficiency (94.4%) for particles and 89.1% for Hep G2 cells were achieved. The majority (>96.6%) of RBCs were removed simultaneously at either case. Since our system only need routine saline solution as buffer, we could completely eliminate any sample preparation steps in our system even at 2× dilution mode, using a saline-preloaded Vacutainer tube to collect clinical sample. No additives will be necessary and thus little contamination. Furthermore, we have successfully demonstrated the isolation of CTCs from clinical sample as a proof of prototype. We observed that the purity of CTCs obtained from our device needs to be further improved to meet the need of various applications. Our simple system permits size-selective isolation without external forces, easy setup and operation, and fast processing. We envision our approach could be a promising powerful technique for a wide range of diagnostic or prognostic applications.

## Materials and Methods

### Device fabrication

Microchannels were fabricated using standard soft lithography. Briefly, we utilized negative resist of SU-8 3025 (MicroChem Corp.) to pattern the microchannels on a 4″ polished silicon wafer by conventional photolithography. Polydimethylsiloxane (PDMS, Down Corning®) was casted on the wafer and peeled after 2 hour curing on 80 °C hotplate. Replicated channels in PDMS were bonded to 1″ × 3″ glass slides (Citotest Labware Manufacturing Co., Ltd) using surface plasma treatment (Harrick Plasma PDC-002). The inlet and outlet ports were punched manually using stainless flat head needles.

### Experimental setup

Samples and buffer solutions were injected into the PDMS device with a syringe pump (NE-4002×, New Era Pump Systems, Inc.) to sustain stable flow rate. The loaded syringe was connected to 1/16” Peek tubings (IDEX Health & Science LLC) using proper fittings (IDEX Health & Science LLC) and then secured to the device inlets. Output of each outlet were collected in 1.5 mL centrifuge tubes. The outlet resistances were carefully tuned for different applications^84^: the resistance ratios of central outlet over single side outlet were 0.597 and 1.29 for whole blood and 2× diluted blood sample, respectively.

The flows of the fluorescently-labeled beads and cells in microchannels were visualized at successive downstream positions using an inverted fluorescence microscope (Leica DMI 4000) equipped with a high-speed EM-CCD camera (iXon ultra 897, Andor Technology Ltd). Analogous to microparticle streak velosimetry (µ-PSV), flowing particles generated streaks across each frame, and we analyzed fluorescent intensities and locations of these particle streaks. Fluorescent, bright-field and phase-contrast images were acquired during experiments. At least 500 frames were obtained and stacked using ImageJ^®^ at each downstream position to improve image contrast. Fluorescence intensity linescans were used in quantitative analyses of focusing quality. Full width at half maximum (FWHM) was used to determine the widths of particle and blood streams in Figs 3 and 4.

### Numerical simulations

Numerical model (Figure S1) was created in CFD-GEOM (ESI Group) with the same layout to the actual device. To reduce the calculation load, we ended the channel at 2 mm downstream length. Unstructured triangle mesh was used and the mesh cell size spanning from 0.02 to 2 µm, resulting in a total of 250,000 cells. Flow module in CFD-ACE+ (ESI Group) was used to model steady state flow. Total flow rate at 225 µL/min and flow rate ratio *α* = 1 were set, which were identical to our optimal experimental conditions. To model blood stream at different dilution factors, we adjusted dynamic viscosity from 4 to 1 mPa-s for the side sample streams and kept the central saline stream unchanged at 1 mPa-s. Densities of the fluids were matched. The convergence criteria was set to 10^−6^.

### Blood sample collection and preparation

Human peripheral blood was obtained from healthy and patient donors after informed consent and according to experimental protocols approved by institutional review board of Sir Run Run Shaw Hospital. Blood was collected in 2 ml Vacutainer (BD). Whole blood was diluted in saline solution to reach different hematocrit/cell concentrations (1×, 2×, 5×, 10×, 20×, 40×, 80× and 200× dilution). Original hematocrit was determined as 45% from blood test report carried out in hospital. Fluorescent 18.7 µm diameter polystyrene particles (Polyscience, Inc.) were spiked into each sample at concentration of ~5.4 × 10^4^ particles/mL.

### Cell culture and staining

HepG2 cells provided by Professor Xiujun Cai at Zhejiang University were cultured in complete growth medium comprised of DMEM (Gibco Cat. No. 11995-065) with 10% (vol/vol) FBS (Gibco Cat. No. 10099-133) and 1% (vol/vol) Penicillin-Streptomycin (Gibco Cat. No. 15140-148) in 25 cm^2^ flask. Cell passages were carried out when the confluency reached 70%–80%. Briefly, cells were rinsed twice with PBS (phosphate-buffered saline) at room temperature after removal of culture media; then 1 ml Trypsin-EDTA solution (Gibco Cat. no. 25300-054) was added and kept in incubator for 4 min to digest the cell layer. Trypisinization was quenched by adding 1 ml complete growth media; cell suspension was transferred into 15 ml conical tube and centrifuged at 1000 rpm for 5 min; cell pellet was re-suspended in saline and cell concentration determined by hemocytometry. A 5 mL of diluted cell suspension at concentration of 5 × 10^4^ cells /mL was aspirated into a new flask for new culture. Culture flask was kept in 37 °C incubator with humidified atmosphere of 5% CO_2_ after spreading the cells evenly by rocking the flask back and forth.

Cells were stained using Hoechst 33258 stain (Molecular Probes) at a final concentration of 2 μg/mL. Cells were incubated in a humidified chamber at 37 °C and 5% CO_2_ for 20–30 min before re-suspending to desired concentration. Blood cells and suspected CTCs were collected from the target outlet and stained immuoselectively. Cells were fixed using 4% formaldehyde for 30 min in exhaust hood and permeablized by 0.2% Triton X-100 (Sigma-Aldrich) for 5 min. The permeablized cells were washed in PBS buffer supplemented with 0.5% BSA. FITC-conjugated pan-cytokeratin (CK) antibody (1:100) and PE-Texas Red conjugated CD45 (1:100) antibody were used successively and allowed to incubate for 30 min. used DAPI for nuclei staining. The stained cells were washed with 1–2 mL of PBS buffer supplemented with 0.5% (wt/vol) BSA for observation under microscope.

### Cell counting

Cell counting was implemented using hemocytometry. Samples collected from each outlet were gently stirred to allow random dispersion. Due to the high concentration of RBCs, we used fluorescently-activated counting, which counted target cells by fluorescent spots under microscope. A 100× dilution of samples was performed before loading sample into hemocytometry for RBC counting due to the high concentration of erythrocytes. This dilution was not necessary for counting fluorescently labeled cells or particles. Images of the counting chamber were taken by CCD camera (bright field for RBCs and fluorescent field for labeled targets) and the counting was performed automatically using ImageJ^®^ “Analyze particle” module with a proper threshold set to discriminated cells. Each sample was counted at least three times to improve accuracy.

Device performance was quantified using the following well-established terms,

$$\begin{array}{c}Efficiency=\frac{Number\,of\,target\,cells\,from\,target\,outlet}{Total\,number\,of\,target\,cells\,from\,both\,outlets}\\ RBC\,rejection\,rate=\frac{Number\,of\,RBCs\,from\,waste\,outlet}{Total\,number\,of\,RBCs\,from\,both\,outlet}\\ Recovery\,rate=\frac{Number\,of\,target\,cells\,from\,target\,outlet}{Number\,of\,target\,cells\,injected}\end{array}$$

### Data availability

Most data are presented in the main text or the supplementary information. All data are available from the corresponding author on reasonable request.

## Electronic supplementary material

Supplementary Information

## References

[1] El-Ali J, Sorger PK, Jensen KF (2006). Cells on chips. Nature.

[2] Mach AJ, Adeyiga OB, Di Carlo D (2013). Microfluidic sample preparation for diagnostic cytopathology. Lab Chip.

[3] May M (2016). Automated sample preparation. Science.

[4] Toner M, Irimia D (2005). Blood-on-a-chip. Annu Rev Biomed Eng.

[5] Miyamoto DT, Sequist LV, Lee RJ (2014). Circulating tumour cells [mdash] monitoring treatment response in prostate cancer. Nat Rev Clin Oncol.

[6] Alix-Panabières C, Pantel K (2014). Technologies for detection of circulating tumor cells: Facts and vision. Lab Chip.

[7] Kaiser J (2010). Medicine. Cancer’s circulation problem. Science.

[8] Nagrath S (2007). Isolation of rare circulating tumour cells in cancer patients by microchip technology. Nature.

[9] den Toonder J (2011). Circulating tumor cells: the Grand Challenge. Lab. Chip.

[10] Kang JH (2012). A combined micromagnetic-microfluidic device for rapid capture and culture of rare circulating tumor cells. Lab Chip.

[11] Chen X (2017). A Simplified Microfluidic Device for Particle Separation with Two Consecutive Steps: Induced Charge Electro-osmotic Prefocusing and Dielectrophoretic Separation. Anal. Chem..

[12] Ren Y (2018). Flexible particle flow‐focusing in microchannel driven by droplet‐directed induced‐charge electroosmosis. Electrophoresis.

[13] Augustsson P, Magnusson C, Nordin M, Lilja H, Laurell T (2012). Microfluidic, label-free enrichment of prostate cancer cells in blood based on acoustophoresis. Anal. Chem..

[14] MacDonald MP, Spalding GC, Dholakia K (2003). Microfluidic sorting in an optical lattice. Nature.

[15] Yu ZT, Aw Yong KM, Fu J (2014). Microfluidic blood cell sorting: now and beyond. Small.

[16] Martel JM, Toner M (2014). Inertial focusing in microfluidics. Annu Rev Biomed Eng.

[17] Huang LR, Cox EC, Austin RH, Sturm JC (2004). Continuous particle separation through deterministic lateral displacement. Science.

[18] McGrath J, Jimeneza M, Bridle H (2014). Deterministic lateral displacement for particle separation: a review. Lab Chip.

[19] Yamada M, Nakashima M, Seki M (2004). Pinched flow fractionation: Continuous size separation of particles utilizing a laminar flow profile in a pinched microchannel. Anal. Chem..

[20] Bhagat AAS, Hou HW, Li LD, Lim CT, Han J (2011). Pinched flow coupled shear-modulated inertial microfluidics for high-throughput rare blood cell separation. Lab Chip.

[21] Yamada M, Seki M (2005). Hydrodynamic filtration for on-chip particle concentration and classification utilizing microfluidics. Lab Chip.

[22] Pamme N (2007). Continuous flow separations in microfluidic devices. Lab. Chip.

[23] Gossett DR (2010). Label-free cell separation and sorting in microfluidic systems. Anal. Bioanal. Chem..

[24] Zhang J (2016). Fundamentals and applications of inertial microfluidics: A review. Lab Chip.

[25] Nam J, Lim H, Kim D, Jung H, Shin S (2012). Continuous separation of microparticles in a microfluidic channel via the elasto-inertial effect of non-Newtonian fluid. Lab Chip.

[26] Liu C (2015). Size-Based Separation of Particles and Cells Utilizing Viscoelastic Effects in Straight Microchannels. Anal. Chem..

[27] Ozkumur E (2013). Inertial focusing for tumor antigen-dependent and -independent sorting of rare circulating tumor cells. Sci. Transl. Med..

[28] Lu X, Xuan X (2015). Elasto-Inertial Pinched Flow Fractionation for Continuous Shape-Based Particle Separation. Anal. Chem..

[29] Stott SL (2010). Isolation of circulating tumor cells using a microvortex-generating herringbone-chip. Proc. Natl. Acad. Sci. USA.

[30] Shevkoplyas SS, Yoshida T, Munn LL, Bitensky MW (2005). Biomimetic autoseparation of leukocytes from whole blood in a microfluidic device. Anal. Chem..

[31] Jain A, Munn LL (2011). Biomimetic postcapillary expansions for enhancing rare blood cell separation on a microfluidic chip. Lab on a chip.

[32] Li X, Chen W, Liu G, Lu W, Fu J (2014). Continuous-flow microfluidic blood cell sorting for unprocessed whole blood using surface-micromachined microfiltration membranes. Lab Chip Miniaturisation Chem. Biol..

[33] Cheng Y, Ye X, Ma Z, Xie S, Wang W (2016). High-throughput and clogging-free microfluidic filtration platform for on-chip cell separation from undiluted whole blood. Biomicrofluidics.

[34] Davis JA (2006). Deterministic hydrodynamics: taking blood apart. Proc. Natl. Acad. Sci. USA.

[35] Choi J, Hyun JC, Yang S (2015). On-chip Extraction of Intracellular Molecules in White Blood Cells from Whole Blood. Sci. Rep..

[36] Park ES (2016). Continuous Flow Deformability-Based Separation of Circulating Tumor Cells Using Microfluidic Ratchets. Small.

[37] Kim B, Choi YJ, Seo H, Shin E, Choi S (2016). Deterministic Migration‐Based Separation of White Blood Cells. Small.

[38] Hou HW (2013). Isolation and retrieval of circulating tumor cells using centrifugal forces. Sci. Rep..

[39] Warkiani ME (2016). Ultra-fast, label-free isolation of circulating tumor cells from blood using spiral microfluidics. Nat. Protoc..

[40] Baskurt OK, Meiselman HJ (2003). Blood Rheology and Hemodynamics. Semin. Thromb. Hemost..

[41] Chien S, Usami S, Taylor HM, Lundberg JL, Gregersen MI (1966). Effects of hematocrit and plasma proteins on human blood rheology at low shear rates. J. Appl. Physiol..

[42] Chien S (1981). Determinants of blood viscosity and red cell deformability. Scand. J. Clin. Lab. Invest..

[43] Merrill EW (1969). Rheology of blood. Physiol. Rev..

[44] Wells RE, Merrill EW (1961). Shear rate dependence of the viscosity of whole blood and plasma. Science.

[45] Thurston GB (1989). Plasma release-cell layering theory for blood flow. Biorheology.

[46] Yang S, Kim JY, Lee SJ, Lee SS, Kim JM (2011). Sheathless elasto-inertial particle focusing and continuous separation in a straight rectangular microchannel. Lab Chip.

[47] Rodd LE, Scott TP, Boger DV, Cooper-White JJ, McKinley GH (2005). The inertio-elastic planar entry flow of low-viscosity elastic fluids in micro-fabricated geometries. J. Non Newtonian Fluid Mech..

[48] Ho BP, Leal LG (1976). Migration of rigid spheres in a two-dimensional unidirectional shear flow of a second-order fluid. J. Fluid Mech..

[49] Villone MM, D’Avino G, Hulsen MA, Greco F, Maffettone PL (2011). Simulations of viscoelasticity-induced focusing of particles in pressure-driven micro-slit flow. J. Non-Newton. Fluid Mech..

[50] Leshansky AM, Bransky A, Korin N, Dinnar U (2007). Tunable nonlinear viscoelastic “focusing” in a microfluidic device. Phys. Rev. Lett..

[51] Del Giudice F (2013). Particle alignment in a viscoelastic liquid flowing in a square-shaped microchannel. Lab Chip.

[52] D’Avino G (2012). Single line particle focusing induced by viscoelasticity of the suspending liquid: Theory, experiments and simulations to design a micropipe flow-focuser. Lab Chip.

[53] Zhou J, Papautsky I (2013). Fundamentals of inertial focusing in microchannels. Lab Chip.

[54] Dai S-, Qi F, Tanner RI (2014). Viscometric functions of concentrated non-colloidal suspensions of spheres in a viscoelastic matrix. J. Rheol..

[55] Lim EJ (2014). Inertio-elastic focusing of bioparticles in microchannels at high throughput. Nat. Commun..

[56] Kim JY, Ahn SW, Lee SS, Kim JM (2012). Lateral migration and focusing of colloidal particles and DNA molecules under viscoelastic flow. Lab Chip.

[57] Kang K, Lee SS, Hyun K, Lee SJ, Kim JM (2013). DNA-based highly tunable particle focuser. Nat Commun.

[58] Chien S (1970). Shear dependence of effective cell volume as a determinant of blood viscosity. Science.

[59] Thurston GB, Henderson NM (2006). Effects of flow geometry on blood viscoelasticity. Biorheology.

[60] Chien S, Usami S, Dellenback RJ, Gregersen MI (1970). Shear-dependent deformation of erythrocytes in rheology of human blood. Am. J. Physiol..

[61] Zhou J, Giridhar PV, Kasper S, Papautsky I (2014). Modulation of rotation-induced lift force for cell filtration in a low aspect ratio microchannel. Biomicrofluidics.

[62] Pries AR, Neuhaus D, Gaehtgens P (1992). Blood viscosity in tube flow: Dependence on diameter and hematocrit. Am J Physiol.

[63] Zhou J, Giridhar PV, Kasper S, Papautsky I (2013). Modulation of aspect ratio for complete separation in an inertial microfluidic channel. Lab Chip.

[64] Leighton D, Acrivos A (1987). The shear-induced migration of particles in concentrated suspensions. J. Fluid Mech..

[65] Eckstein EC, Bailey DG, Shapiro AH (1977). Self-diffusion of particles in shear flow of a suspension. J. Fluid Mech..

[66] Kim W (2010). Particle image velocimetry of the blood flow in a micro-channel using the confocal laser scanning microscope. Journal of the Optical Society of Korea.

[67] Bossis G, Brady J (1987). Self‐diffusion of Brownian particles in concentrated suspensions under shear. J. Chem. Phys..

[68] Segré G, Silberberg A (1961). Radial particle displacements in poiseuille flow of suspensions. Nature.

[69] Di Carlo D (2009). Inertial microfluidics. Lab Chip.

[70] Di Carlo D, Irimia D, Tompkins RG, Toner M (2007). Continuous inertial focusing, ordering, and separation of particles in microchannels. Proc. Natl. Acad. Sci. USA.

[71] Bhagat AAS (2010). Microfluidics for cell separation. Med. Biol. Eng. Comput..

[72] Zhang J, Yan S, Li W, Alici G, Nguyen N (2014). High throughput extraction of plasma using a secondary flow-aided inertial microfluidic device. RSC Adv..

[73] Madanshetty SI, Nadim A, Stone H (1996). Experimental measurement of shear‐induced diffusion in suspensions using long time data. Phys. Fluids.

[74] Huang P, Joseph D (2000). Effects of shear thinning on migration of neutrally buoyant particles in pressure driven flow of Newtonian and viscoelastic fluids. J. Non Newtonian Fluid Mech..

[75] Tehrani M (1996). An experimental study of particle migration in pipe flow of viscoelastic fluids. J. Rheol.

[76] Thurston GB (1972). Viscoelasticity of human blood. Biophys. J..

[77] Thurston GB (1976). The viscosity and viscoelasticity of blood in small diameter tubes. Microvasc. Res..

[78] Geislinger, T. M., Stamp, M. E. M., Wixforth, A. & Franke, T. Hydrodynamic and label-free sorting of circulating tumor cells from whole blood. *Appl*. *Phys*. *Lett*. **107** (2015).

[79] Loutherback, K. *et al*. Deterministic separation of cancer cells from blood at 10 mL/min. *AIP Advances* **2** (2012). 10.1063/1.4758131 PMC3477176 23112922

[80] D’Silva J, Austin RH, Sturm JC (2015). Inhibition of clot formation in deterministic lateral displacement arrays for processing large volumes of blood for rare cell capture. Lab on a Chip.

[81] Zhang J (2014). Inertial particle separation by differential equilibrium positions in a symmetrical serpentine micro-channel. Sci. Rep.

[82] Lee MG, Shin JH, Bae CY, Choi S, Park J- (2013). Label-free cancer cell separation from human whole blood using inertial microfluidics at low shear stress. Anal. Chem..

[83] Xu W (2011). Isolation of circulating tumor cells in patients with hepatocellular carcinoma using a novel cell separation strategy. Clin. Cancer Res..

[84] Tu C (2017). A flexible cell concentrator using inertial focusing. Biomed. Microdevices.

