# Supplementary material for: Isolation of cells from whole blood using shear-induced diffusion
Source: Sci Rep. 2018 Jun 20;8:9411. doi: 10.1038/s41598-018-27779-2 (PMC6010421; doi:10.1038/s41598-018-27779-2)
Supplement: Supplementary file 1 — Supplementary Information [file 41598_2018_27779_MOESM1_ESM.pdf]

Supplementary Information

Title: Isolation of cells from whole blood using shear-induced diffusion

Authors: *Jian Zhou*, Chunlong Tu, Yitao Liang, Bobo Huang, Yifeng Fang, Xiao Liang, Ian Papautsky and Xuesong Ye**

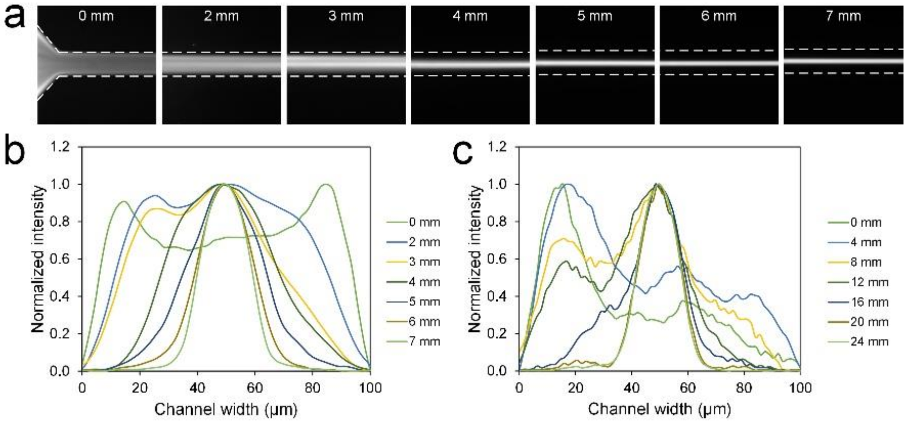

Figure S1 Particle focusing dynamics within Newtonian fluid and non-Newtonian blood (2× dilution, images in Figure 2a). (a) Focusing of 18.7 μm diameter particles dispersed in deionized water within 8 mm downstream length in a 100 μm × 50 μm channel at *Re* = 30. (b) Intensity profiles as a function of downstream position corresponding to part (a). (c) Intensity profiles as a function of downstream position of particle migration within non-Newtonian blood sample (2× dilution) in 100 μm × 50 μm channel at *Re* ≈ 30.

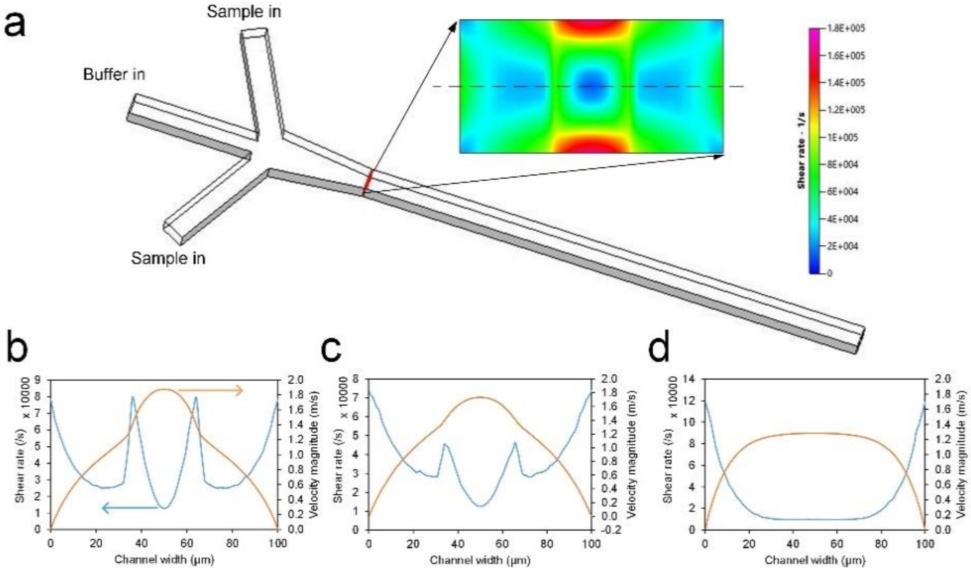

Figure S2 Numerical modeling of co-flow system. (a) Numerical 3D modeling (ESI Group ACE+) of our co-flow channel and the profiles of velocity and shear rate at the beginning of the main channel (dashed line) for three viscosity ratios of sample over buffer: (b) 4, (c) 2 and (d) 1.

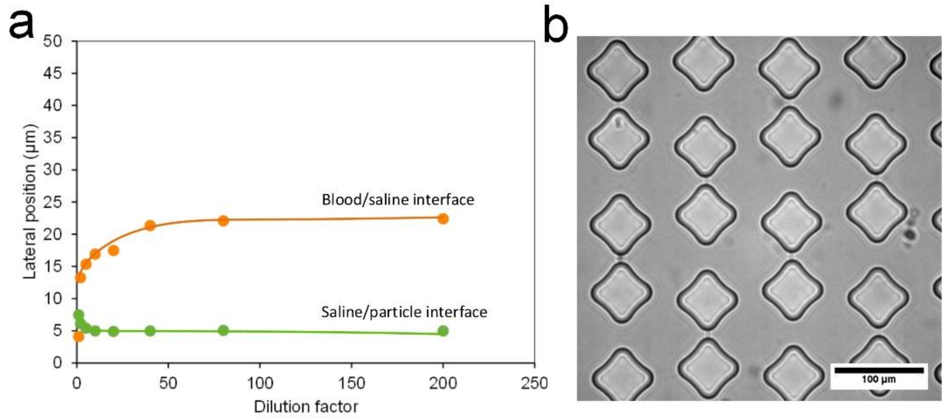

Figure S3 (a) Replot of interface positions as a function of dilution factor in Figure 2c. Lateral position at 0 µm represents channel center axis. (b) Micropost array used to capture target cells separated in the upstream co-flow system.
